# Optimized Variational Mode Decomposition and Permutation Entropy with Their Application in Feature Extraction of Ship-Radiated Noise

**DOI:** 10.3390/e23050503

**Published:** 2021-04-22

**Authors:** Dongri Xie, Shaohua Hong, Chaojun Yao

**Affiliations:** 1 Sichuan Aerospace Electronic Equipment Research Institute, Chengdu 610100, China; 23320171153152@stu.xmu.edu.cn; 2 School of Informatics, Xiamen University, Xiamen 316005, China; hongsh@xmu.edu.cn; 3 Shenzhen Research Institute of Xiamen University, Shenzhen 518057, China

**Keywords:** ship-radiated noise, variational mode decomposition, permutation entropy, reverse weighted permutation entropy, Spearman correlation coefficient

## Abstract

The complex and changeable marine environment surrounded by a variety of noise, including sounds of marine animals, industrial noise, traffic noise and the noise formed by molecular movement, not only interferes with the normal life of residents near the port, but also exerts a significant influence on feature extraction of ship-radiated noise (S-RN). In this paper, a novel feature extraction technique for S-RN signals based on optimized variational mode decomposition (OVMD), permutation entropy (PE), and normalized Spearman correlation coefficient (NSCC) is proposed. Firstly, with the mode number determined by reverse weighted permutation entropy (RWPE), OVMD decomposes the target signal into a set of intrinsic mode functions (IMFs). The PE of all the IMFs and SCC between each IMF with the raw signal are then calculated, respectively. Subsequently, feature parameters are extracted through the sum of PE weighted by NSCC for the IMFs. Lastly, the obtained feature vectors are input into the support vector machine multi-class classifier (SVM) to discriminate various types of ships. Experimental results indicate that five kinds of S-RN samples can be accurately identified with a recognition rate of 94% by the proposed scheme, which is higher than other previously published methods. Hence, the proposed method is more advantageous in practical applications.

## 1. Introduction

Before the rise of artificial neural networks, the recognition of warships in military confrontations mainly relied on sonar soldiers. The recognition performance would fluctuate with changes in the status, experience, and knowledge reserves of technicians, making the results somewhat unstable. With the continuous development of science and technology, the recognition accuracy of underwater acoustic target has been improved to a great extent with the technicians assisted by machine learning. The line spectrum com-ponents in ship-radiated noise (S-RN) signals carrying rich information that characterizes the target, such as speed, tonnage, and type, provides the main basis for target classification [1]. Nevertheless, attributed to the interference of marine environmental noise, along with the time-varying nature of underwater acoustic channel, feature extraction of S-RN becomes increasingly difficult.

Despite the satisfactory performance of traditional signal analysis methods, they fade under the non-linear, non-stationary and non-Gaussian property of underwater acoustic signals. For example, Fourier transform (FT) [2] provides a global description of the overall singularity of the target signal, but it fails to point out the local contribution to the overall singularity, thereby making it unable to locate the specific moment when the mutation occurs. Short-time Fourier transform (STFT) [3] is more effective in a given time interval and frequency interval, but a uniform window function is utilized for all frequency and then the resolution remains unchanged. Therefore, once the window function selected, short-term high-frequency information cannot be refined to an arbitrarily small local time, it thus cannot sensitively reflect signal changes. Wavelet analysis can reflect the requirements of different frequency and have good adaptability, which is suitable for processing sudden changes or isolated singular signals. However, in the process of using wavelet transform (WT) [4] to detect the sudden change of the signal, the number of decomposition layers, wavelet function and noise interference will all have a certain impact on the detection result.

Fortunately, empirical mode decomposition (EMD) [5] can be able to address the is-sues of FT and WT. It has been extensively applied in marine [6], atmospheric [7,8], and mechanical fault diagnosis [9,10,11], etc. Essentially different from FT and WT methods based on a priori assumption of harmonic basis function and wavelet basis function, EMD adaptively decomposes the target signal into several intrinsic mode functions (IMFs) according to the time scale of data itself. Furthermore, EMD is theoretically suitable for analysis of any type of time series, especially in processing non-linear and non-stationary data, which has more advantages than previous smoothing methods. However, mode aliasing and end effect greatly restrict the further development of EMD. To conquer the issue in EMD, ensemble empirical mode decomposition (EEMD) [12] is proposed on the basis of EMD. In the decomposition process, one or more groups of white noise are added to the target signal, thus mode aliasing and end effect can be effectively suppressed. However, residual noise will inevitably be introduced during EEMD processing. As another breakthrough in the field of signal processing, variational mode decomposition (VMD) [13] is also applicable to non-stationary and nonlinear signals. By assuming that each mode is a narrow-band signal compact at a center frequency, VMD employs iterative search for the optimal solution of the variational model to calculate the mode and corresponding center frequency. VMD has successfully remedied the deficiency of EMD and EEMD, but the mode number and quadratic penalty term need to be set in advance.

In general, signal decomposition methods alone cannot complete feature extraction. A noisy signal can be decomposed by a signal decomposition algorithm into noise, noise-dominated IMFs, and pure IMFs. Among them, noise IMFs are usually undesirable. Hence, only when the noise is excluded from feature extraction, can the classification ac-curacy be well guaranteed. One way to tackle this issue is via entropy algorithms. As one of the important theories in the field of nonlinear dynamics, entropy is a quantitative indicator for complexity of time series. In the past few decades, an increasing number of entropy methods have been emerging, such as fuzzy entropy (FE) [14], sample entropy (SE) [15], approximate entropy (AE) [16], and permutation entropy (PE) [17], etc. Since it was proposed by Bandt et al. in 2002, PE has attracted extensive attention from scholars. Explosively, PE begun to spring up in multiple fields, such as financial activity forecasting [18,19], agricultural product research [20], EEG signal analysis [21,22], mechanical fault diagnosis [23,24], and underwater acoustic signal processing [25,26,27,28], etc. Unfortunately, the inherent flaw of PE lies in disregard for the amplitude of time series, as it only focuses on the ordinal patterns for neighboring vectors. As the complementary versions of PE, the weighted permutation entropy (WPE) [29] and amplitude-aware permutation entropy (AAPE) [30] achieve stronger recognition ability for various signals than PE by assigning higher weights to sensitive patterns. Reverse permutation entropy (RPE) showing the opposite trend to the traditional entropy methods, quantifies the distance from Gaussian white noise. Meanwhile, the better stability of RPE than that of PE in terms of data length has been validated in [31]. Besides, RPE has never been used as the feature vector of underwater acoustic targets yet. On the basis of RPE and WPE, reverse weighted permutation entropy (RWPE) inherits the merits of both RPE and WPE in a proper manner, which concerns the amplitude and distance information of time series. The experimental results in [32] have demonstrated the good stability and powerful recognition ability of noise for RWPE.

In recent years, VMD combined with various entropy algorithms and its extended version have been utilized in full swing for underwater acoustic signal processing. In [27], based on VMD, the multi-scale permutation entropy (MPE) of IMF with the highest energy was extracted, achieving a classification accuracy of 94%. In [33], a finite number of IMFs were firstly decomposed by VMD of S-RN signals. The IMF with the smallest fluctuation-based dispersion entropy (FDE) difference with the original signal was regarded as the sensitive IMF, whose FDE was input into self-organizing map classifier to reach an identification rate of 97.5%. In [25], the S-RN signals were firstly decomposed by enhanced VMD into several IMFs. Then the WPE of all the IMFs and their Pearson correlation coefficients (PCCs) with the target signal were calculated, respectively. The sensitive IMFs were screened out by the maximum variance of WPE obtained. Finally, the sum of PE weighted by the normalized PCC for the sensitive IMFs was regarded as the feature vector fed into the SVM, which has been demonstrated to yield better recognition performance. Despite the impressive results of the above methods, there still exist some points to be urgently rectified: (1) the mode number of VMD in [27,33] is consistent with the decomposition results of EMD, which does not make sense in theory; (2) in [33], the noise IMF inadequately characterizing the original signal may be locked by means of the selection method for the sensitive IMF. As we know, the S-RN signal contains rich low-frequency line spectrum components in the range of 0–100 Hz, carrying abundant information about ships, and should not be excluded. Thus, there obviously lacks persuasiveness in [33]. To solve these problems, a new feature extraction technique for S-RN signals based on optimized VMD (OVMD), PE, and normalized Spearman CC (NSCC) is put forward in this paper. Firstly, with the mode number determined by RWPE, a set of IMFs are decomposed by OVMD of the target signal. Then, the PE of all the IMFs and SCC between each IMF with the raw signal are calculated, respectively. Finally, the feature vectors extracted through the sum of PE weighted by NSCC for the IMFs are fed into classifier to realize the classification of S-RN samples.

The main innovations and contributions of this paper are summarized as follows:

(1) VMD is proposed to conquer the mode number issue for VMD, where RWPE is utilized to lock the mode number. Experimental results on sinusoidal signals have proved the better decomposition performance of OVMD than that of EMD and EEMD.
(2) A novel ship-radiated noise feature extraction technique based on OVMD, PE, and NSCC is put forward. The classification results of five kinds of measured S-RN samples indicate that the proposed method is obviously superior to the existing methods with higher recognition rate.

The structure of the paper is organized below: Section 2 is the background. A brief description of the proposed technique is presented in Section 3. The proposed method is utilized for analysis of simulation signals in Section 4. In Section 5, the measured S-RN data are used to test the performance of the proposed technique. Finally, the conclusion is drawn in Section 6.

## 2. Background

### 2.1. Variational Mode Decomposition (VMD)

VMD is an adaptive, completely non-recursive mode variational and signal processing method [13]. It overcomes the problem of end effect and mode aliasing in EMD, and has a more solid mathematical theoretical foundation. The essence of VMD is to construct and solve variational problems.

Given target signal *f*(t), the constraint variational expression is given by

(1) $$\min_{\left\{u_{k},w_{k}\right\}}\left\{\sum_{k}\|\partial_{t}\left[\left(\delta \left(t\right)+j/\pi t\right)*u_{k}\left(t\right)\right]e^{-jw_{k}t}{\|}_{2}^{2}\right\}s.t.\sum_{k=1}^{K}u_{k}(t)=f\left(t\right)$$

where *K* is the mode number; $\left\{u_{k}\right\}$, $\left\{w_{k}\right\}$ are the *k*-th mode and its center frequency, respectively; δ(t) is the Dirac function, ∂(⋅) is partial derivative, and * means the convolution operation.

To solve the variational problem in Equation (1), the Lagrange multiplication operator λ is introduced and the constrained variational problem is transformed into an unconstrained variational problem. That is, the augmented Lagrange expression is achieved by

(2) $$L\left(\left\{u_{k}\right\},\left\{w_{k}\right\},\lambda \right)=\alpha \sum_{k}\|\partial_{t}\left[\left(\partial_{t}+j/\pi t\right)*u_{k}\left(t\right)\right]e^{-jw_{k}t}{\|}_{2}^{2}+{\left\|f(t)-\sum_{k}u_{k}(t)\right\|}_{2}^{2}+\langle \lambda (t),f(t)-\sum_{k}u_{k}(t)\rangle$$

where α is the quadratic penalty term.

Each mode and corresponding center frequency are calculated by employing alternating direction multipliers, combining Paseval’s theorem and Fourier transform (FT). $\left\{w_{k}\right\}$, $\left\{u_{k}\right\}$, and λ can be finally updated as follows:

(3) $${\hat{u}}_{k}^{n+1}(w)=\frac{\hat{f}(w)-\sum_{i\neq k}{\hat{u}}_{i}(w)+\hat{\lambda }(w)/2}{1+2\alpha {\left(w-w_{k}\right)}^{2}}$$

(4) $$w_{k}^{n+1}=\frac{\int_{0}^{\infty }w{\left|u_{k}(w)\right|}^{2}dw}{\int_{0}^{\infty }{\left|{\hat{u}}_{k}(w)\right|}^{2}dw}$$

(5) $${\hat{\lambda }}^{n+1}(w)={\hat{\lambda }}^{n}(w)+\varepsilon (\hat{f}(w)-\sum_{k}{\hat{u}}_{k}^{n+1}(w))$$

where the noise tolerance ε meets the fidelity requirements of signal decomposition, and ${\hat{u}}_{k}^{n+1}(w)$, $u_{k}(w)$, $\hat{f}(w)$ and $\hat{\lambda }(w)$ correspond to the FT of ${\hat{u}}_{k}^{n+1}(t)$, $u_{k}(t)$, $\hat{f}(t)$, and $\hat{\lambda }(t)$, respectively. In summary, the decomposition process of VMD is briefly summarized as follows:

Step 1: Initialize $u_{k}^{1}$, $w_{k}^{1}$, $\lambda^{1}$ and maximum number of iterations N;

Step 2: Update ${\hat{u}}_{k}$ and $w_{k}$ using Equations (3) and (4);

Step 3: Update λ based on Equation (5);

Step 4: For convergence accuracy *a* > 0, if the convergence condition $\sum_{k}{\left\|{\hat{u}}_{k}^{n+1}-{\hat{u}}_{k}^{n}\right\|}_{2}^{2}/{\left\|{\hat{u}}_{k}^{n}\right\|}_{2}^{2}<a$ is not satisfied, go to step 2; otherwise terminate the iteration and output ${\hat{u}}_{k}$ and $w_{k}$.

### 2.2. Permutation Entropy (PE)

The application of PE in this paper benefits by its being conceptually simple, computationally fast, as well as better stability. For time series {X(i),i=1,2,⋯,n}, embedding dimension m, and time delay τ, the calculation steps for PE are epitomized as the steps below [17]:

The time series can be reconstructed in phase space as:

(6) $$\left[\begin{array}{cccc} x\left(1\right) & x(1+\tau ) & \cdots & x\left(1+\left(m-1\right)\tau \right)\\ \vdots & \vdots & & \vdots \\ x\left(j\right) & x(j+\tau ) & \cdots & x\left(j+\left(m-1\right)\tau \right)\\ \vdots & \vdots & & \vdots \\ x\left(K\right) & x(K+\tau ) & \cdots & x\left(K+\left(m-1\right)\tau \right) \end{array}\right],K=n-\left(m-1\right)\tau$$.

Arrange the elements in *j*-th row in ascending order according to their value:

(7) $$x(i+(j_{1}-1)\tau )\leq x(i+(j_{2}-1)\tau )\leq \cdots \leq x(i+(j_{m}-1)\tau )$$

In the case of two equal elements:

(8) $$x(i+(j_{1}-1)\tau )=x(i+(j_{2}-1)\tau )$$

These two elements are rearranged as:

(9) $$x(i+(j_{1}-1)\tau )\leq x(i+(j_{2}-1)\tau )\;(j_{1}\leq j_{2})$$

Consequently, for each row vector in the reconstruction matrix, a set of symbols can be obtained:

(10) $$S\left(l\right)=\left(j_{1},j_{2},\cdots ,j_{m}\right),\;l=1,2,\cdots ,k,\;and\;k\leq m!$$

The probability of each symbol sequence is $P_{1},P_{2},\cdots ,P_{k}$, the PE can be finally calculated as:

(11) $$H_{p}(m)=-{\left(\ln m!\right)}^{-1}\sum_{g=1}^{k}P_{g}\ln P_{g}$$

The change in $H_{p}$ reflects and magnifies the minute changes in the time series. The smaller the value of $H_{p}$, the more regular the time series; on the contrary, the closer the time series is to random.

### 2.3. Reverse Weighted Permutation Entropy (RWPE)

As a fusion of weighted permutation entropy (WPE) and RPE, reverse weighted permutation entropy (RWPE) enjoys a powerful advantage in detection of signal mutation and recognition of noise [26,32]. In the RWPE algorithm, for time series {X(i),i=1,2,⋯,n}, given embedding dimension *m* and time delay τ, the weight $w_{j}$ of the embedding vector *X_i_* can be calculated as:

(12) $$w_{j}=\frac{1}{m}{\sum_{k=1}^{m}\left[x_{j+\left(k-1\right)\tau }-{\bar{X}}_{j}^{m,\tau }\right]}^{2}$$

(13) $${\bar{X}}_{j}^{m,\tau }=\frac{1}{m}\sum_{k=1}^{m}x_{j+\left(k+1\right)\tau }$$

Thus, the weighted relative frequency is expressed as:

(14) $$p_{w}\left(\pi_{i}^{m,\tau }\right)=\frac{\sum j\leq N^{1_{u:\;\mathrm{type}\left(u\right)=\pi_{i}}\left({\bar{X}}_{j}^{m,\tau }\right)w_{j}}}{\sum j\leq N^{1_{u:\;\mathrm{type}\left(u\right)\;\in \;\Pi }\left({\bar{X}}_{j}^{m,\tau }\right)w_{j}}}$$

where $\pi_{i}^{m,\tau }$ is one of the m! distinct symbols; $1_{I}\left(u\right)$ means the indicator function of set I defined as $1_{I}\left(u\right)=1$ if u∈I, else $1_{I}\left(u\right)=0$.

RWPE is the pointer to distance with Gaussian white noise. In this case, RWPE is finally defined as:

(15) $$H_{RWPE}\left(m,\tau \right)={\sum_{i:\pi_{i}^{m,\tau }\in \Pi }\left(p_{w}\left(\pi_{i}^{m,\tau }\right)-\frac{1}{m!}\right)}^{2}={\sum_{i:\pi_{i}^{m,\tau }\in \Pi }\left(p_{w}\left(\pi_{i}^{m,\tau }\right)\right)}^{2}-\frac{1}{m!}$$

The value range of RWPE is 0 to 1. Contrary to traditional entropy algorithms, a smaller RWPE means a more random time series, and vice versa. In this article, for all calculations involving entropy, as in [17], the embedding dimension and time delay are uniformly set to four and one, respectively.

## 3. The Proposed Feature Extraction Technique

**Figure 1:**
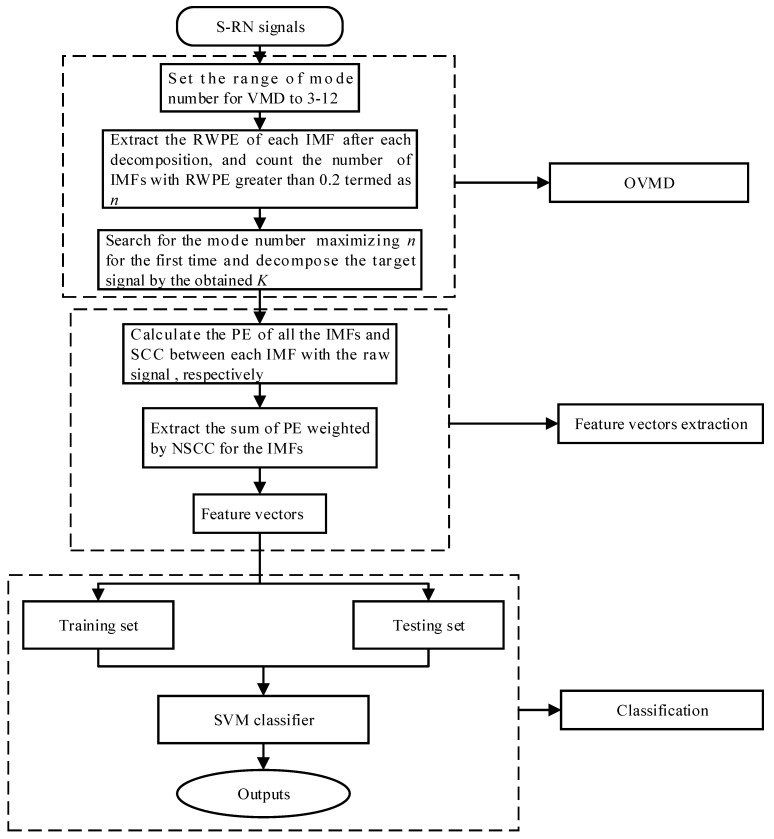
The flow chart of the proposed technique.

This paper proposes a novel feature extraction technique for S-RN signals integrating OVMD, PE, and normalized spearman correlation coefficient (NSCC). The flow chart of the proposed scheme is depicted in Figure 1. The basic steps of the scheme are summarized as follows:

Step 1: Set the range of mode number *K* to 3–12 and decompose the target signal;

Step 2: Calculate the RWPE of each IMF. Subsequently, count the number of IMFs with RWPE greater than 0.2 after each decomposition termed as *n*;

Step 3: Decompose the target signal using the mode number maximizing *n* for the first time;

Step 4: Extract the PE of all the IMFs and their NSCCs with the raw signal;

Step 5: Calculate the sum of PE weighted by NSCC for the IMFs;

Step 6: Divide the data set into training set for training the classifier according to a proper proportion, and the remaining for testing it;

Step 7: Finally, the testing data are fed into the SVM multi-class classifier to complete recognition of various types of S-RN samples.

## 4. Analysis of Simulation Signals

### 4.1. Property Analysis of RWPE

The less sensitivity of PE to data length has been discussed in study [26], we won’t explore it in this paper. After this, we will make a comparison for the signal-recognized ability of PE, WPE, AAPE, RPE and RWPE. To this end, we construct a standard Gaussian white noise series with pulse sequence added, and the data length is set to 5000. We then calculate the above five entropy methods using a window with length of 500 and sliding step of 50.

**Figure 2:**
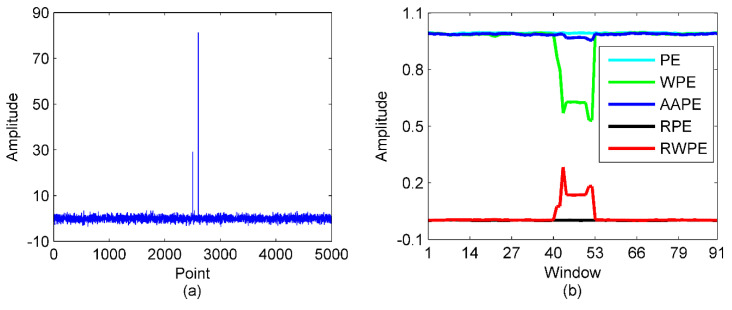
The time−domain waveform of Gaussian white noise with pulse sequence added and calculation results for PE, WPE, AAPE, RPE and RWPE; (**a**) the Gaussian white noise series with pulse sequence added; (**b**) the calculation results.

Figure 2 are the time-domain waveform of the constructed signal and calculation results. As illustrated in Figure 2, there appear to be no difference in the mutation region with other region for PE and RPE, this can be explained by the ignorance of amplitude, but only comparison of ordinal patterns. Rather, attributed to the weight assigned to sensitive patterns, the value of AAPE in the mutation region start to decrease, which is significantly different from other regions. In marked contrast, both WPE and RWPE have shown significant changes in the mutation area, indicating an exceedingly good recognition ability for different signals.

For further quantitative comparison, Table 1 presents the ratio of the maximum and minimum values of the average values for the above five entropies in the mutation region and other regions. It can be observed from Table 1 that the value of RWPE in the mutation area changes most significantly with the obviously larger mutation rate than others. Hence, RWPE is chosen for the calculation candidate of mode number for VMD in this article.

### 4.2. OVMD of Sinusoidal Signals

The mode number K set in advance plays a decisive role in influence on the decomposition accuracy of VMD. If K is too small, useful low-frequency components cannot be completely recovered from the signal submerged by noise, that is, the so-called under-decomposition. However, too large K will cause over-decomposition to occur, that is to say, in addition to undesired IMFs being generated, concurrently, the computational complexity will also be dramatically increased. In view of this, the balance between data recovery and time cost is particularly interesting concerning VMD. Study in [27,33], the decomposition result of EMD is used as a reference for VMD, which is obviously short of reasonable mathematical foundation, as well as may result in over-decomposition. Besides, considering the results of VMD, with the increase of K, low-frequency IMFs are gradually being discovered and become cleaner. When K increases to a certain critical point, the low-frequency components have been completely reproduced, and their complexity will also converge to a certain value. When K continues to increase, the contribution to the decomposition accuracy is negligible except for the significant increase in computational complexity. Inspired by the analysis, given the high sensitivity of RWPE to noise, it is introduced for K selection in VMD (see Section 3).

Subsequently, we will make a specific discussion on how to choose an appropriate RWPE threshold. Due to the single frequency nature of intrinsic mode functions (IMF) by VMD, three sinusoidal signals are utilized for the selection of RWPE threshold. The three sinusoidal signals are given as follows:

(16) $$\left\{ \begin{array}{l} f_{1}\left(t\right)=\cos\left(50\pi t\right)\\ f_{2}\left(t\right)=\cos\left(100\pi t\right)\\ f_{3}\left(t\right)=\cos\left(160\pi t\right) \end{array}\right.$$

where the sampling frequency and the data length are set to 5 kHz and 5000, respectively.

**Figure 3:**
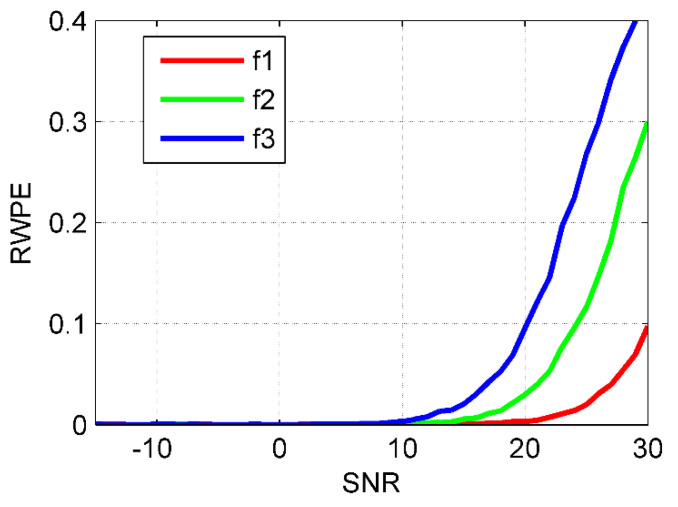
The RWPE for the sinusoidal signals under different SNR.

The RWPE of them under the signal-to-noise ratio (SNR) range of −15–30 dB is shown in Figure 3. As indicated in Figure 3, when the threshold of RWPE is set to be greater than 0.2, the corresponding SNR of the three signals is greater than 20 dB, and their complexity is drastically reduced. In this situation, the signal can be considered approximately pure with the noise being negligible compared to it. Therefore, as K increases, in the case of all the reproduced low-frequency IMFs whose RWPE greater than 0.2 for the first time, the corresponding K is considered to be the optimal mode number.

**Figure 4:**
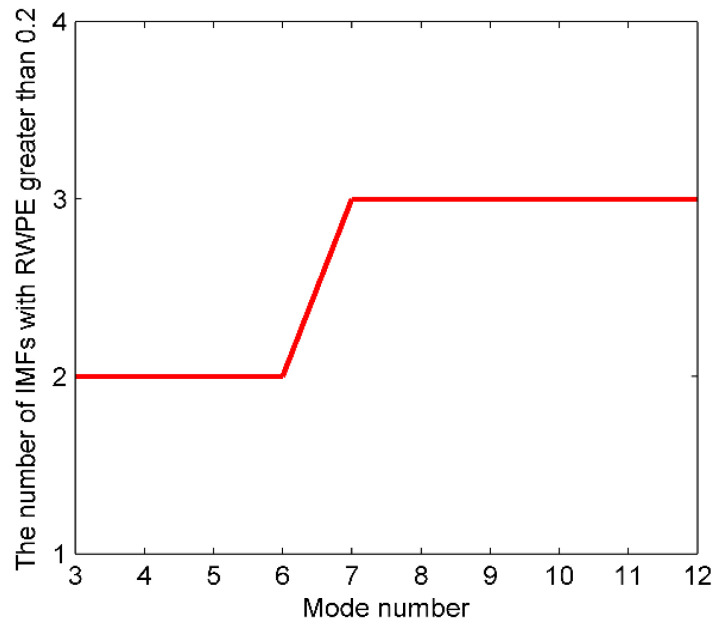
The number of IMFs greater than 0.2 versus the mode number K.

As is well known to us, S-RN signals contain rich line spectrum components being one of the key parameters for underwater acoustic target recognition. Accordingly, in this paper, a composite signal composed of several sinusoidal signals is randomly constructed to validate the performance of the proposed method. The simulation signals are as follows:

(17) $$\left\{ \begin{array}{l} f_{1}\left(t\right)=\cos\left(10\pi t\right)\\ f_{2}\left(t\right)=\cos\left(100\pi t\right)\\ f_{3}\left(t\right)=\cos\left(200\pi t\right)\\ f\left(t\right)=f_{1}\left(t\right)+f_{2}\left(t\right)+f_{3}\left(t\right)+\eta \end{array}\right.$$

where the sampling frequency and the data length are set to 1 kHz and 5000, respectively. η represents the standard Gaussian white noise. As the mode number of VMD is less than that of EEMD, we then set the range of K to 3–12, and count the number of IMFs whose RWPE greater than 0.2 after each decomposition. The number of IMFs greater than 0.2 versus the mode number K is presented in Figure 4.

It can be concluded from Figure 4 that the K maximizing *n* for the first time is 7, thus, 7 is considered as the optimal mode number for VMD. In order to enhance persuasiveness, Table 2 and Table 3 list the center frequency and RWPE of IMFs after each decomposition, respectively.

**Figure 5:**
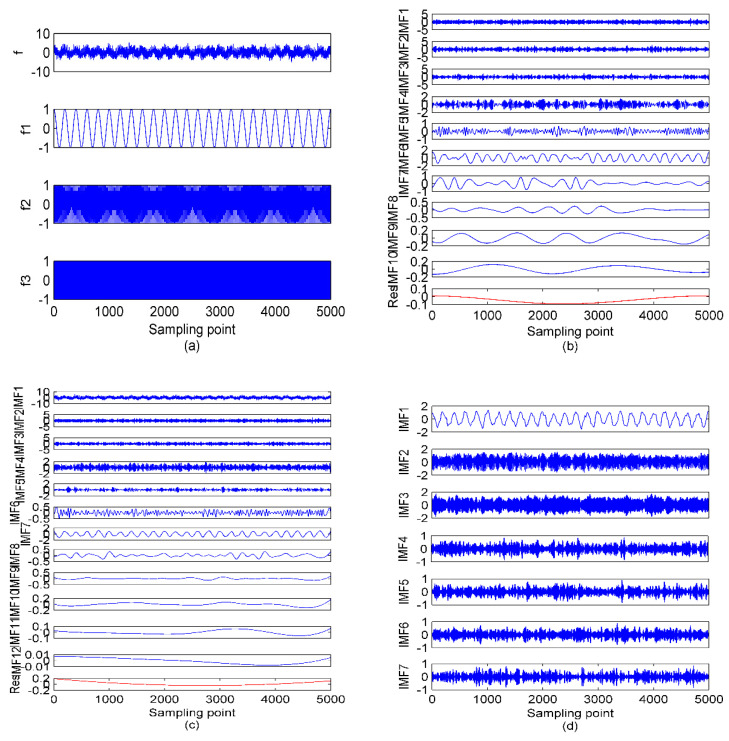
The time−domain waveforms of simulation signals and results of EMD, EEMD, and OVMD; (**a**) the simulation signals; (**b**) EMD; (**c**) EEMD; (**d**) OVMD.

According to Table 2, when K is 3–6, the components corresponding to the simulation signals have not been completely reproduced yet; when K reaches 7, the first three IMFs correspond to the raw signals exactly; when K is more than 8, despite the fully decomposed components, more high-frequency noise is also generated. As for Table 3, when K is 3–6, the number of IMFs with RWPE greater than 0.2 remains 2; when K is 7, the number increases to 3, and the number continues to be 3 when K is more than 7. In this case, 7 is exactly the optimum mode number. Furthermore, with the K increased, the RWPE of useful components are increasing regularly and gradually converging to a fixed value. While the noise IMFs are not the case, thereby scientifically validating the rationality of employing RWPE threshold to lock the mode number for VMD. The time-domain waveforms of simulation signals, along with the decomposition results of EMD, EEMD, and OVMD are shown in Figure 5.

As illustrated in Figure 5, a certain degree of mode aliasing occurs in EMD and EEMD. In contrast, the decomposition performance of OVMD is better than that of EMD and EEMD without this phenomenon. In order to make the decomposition accuracy of OVMD prominent, the mean absolute error (MAE) between the center frequency of IMFs by EMD, EEMD, and OVMD with that of corresponding simulation signals are listed in Table 4. Table 4 shows that the maximum MAE of EMD means poor decomposition performance, while the decomposition performance of EEMD has been improved to a certain extent with a smaller MAE than EMD. Compared with EMD and EEMD, OVMD has revealed the best decomposition performance with the smallest MAE. Therefore, OVMD is superior to EMD and EEMD in terms of recovering the desired components from the signal masked by noise.

## 5. Classification of Measured S-RN Samples

### 5.1. OVMD of S-RN Signals

**Figure 6:**
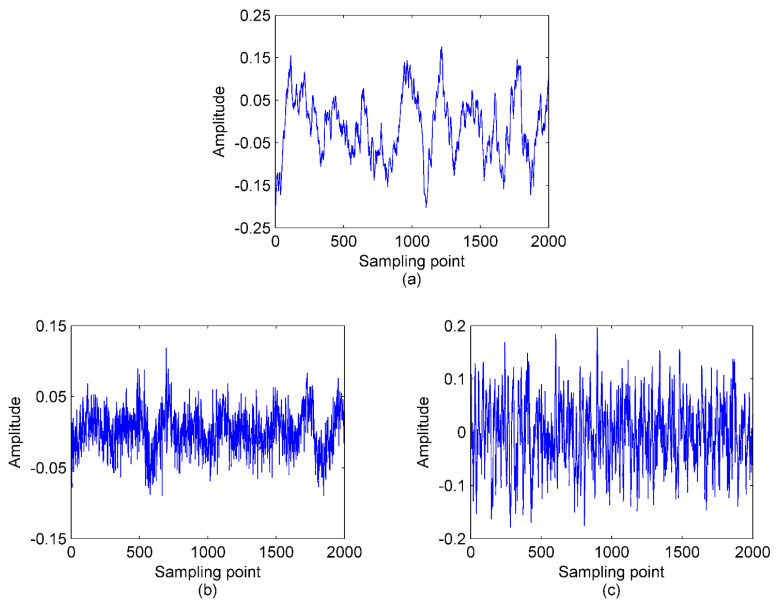

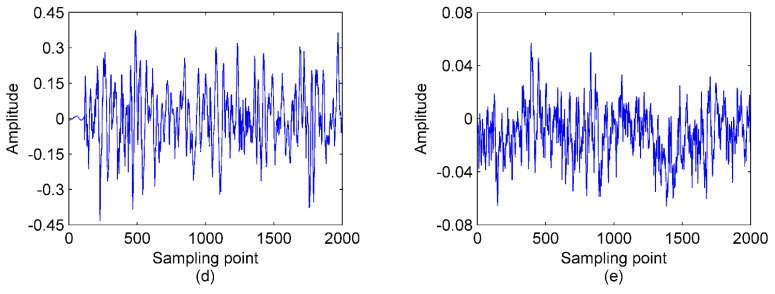
The time−domain waveforms for the three normalized S−RN signals; (**a**) Ship I signal; (**b**) Ship II signal; (**c**) Ship III signal; (**d**) Ship IV signal; (**e**) Ship V signal.

**Figure 7:**
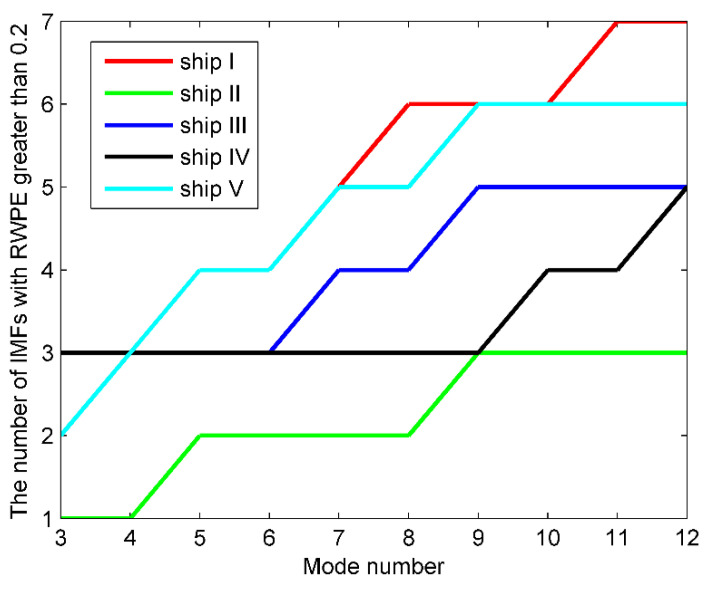
The number of IMFs greater than 0.2 versus mode number for the five types of signals.

In this paper, five types of real measured S-RN samples are randomly selected from a data set in [34], namely, Ship I, Ship II, Ship III, Ship IV, and Ship V. There are 50 randomly selected samples from each category for analysis. The data points are set to 2000. The time-domain waveforms of the five normalized signals are shown in Figure 6 and Figure 7 depicts the results of using the proposed method to lock the mode number of VMD for the five types of signals.

**Figure 8:**
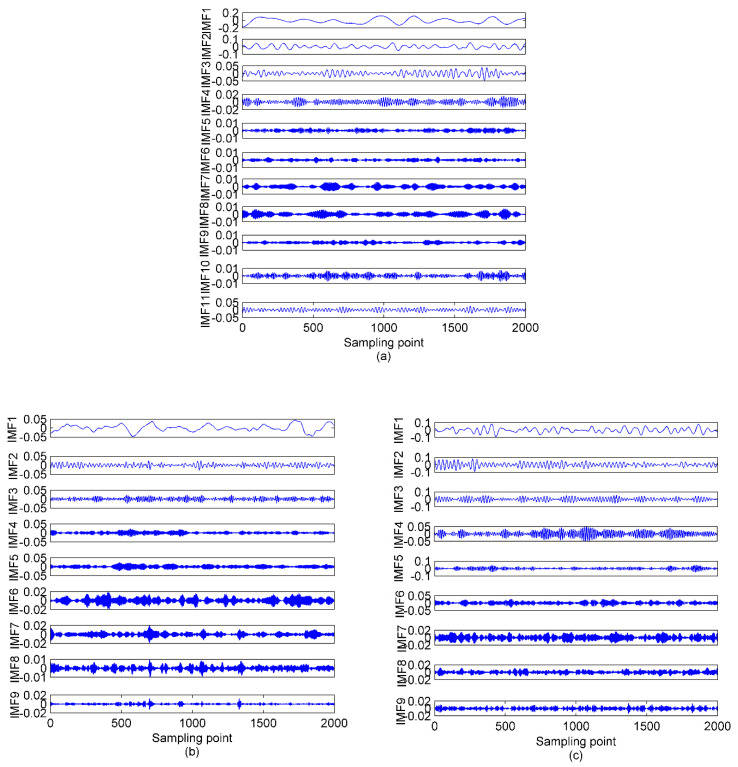

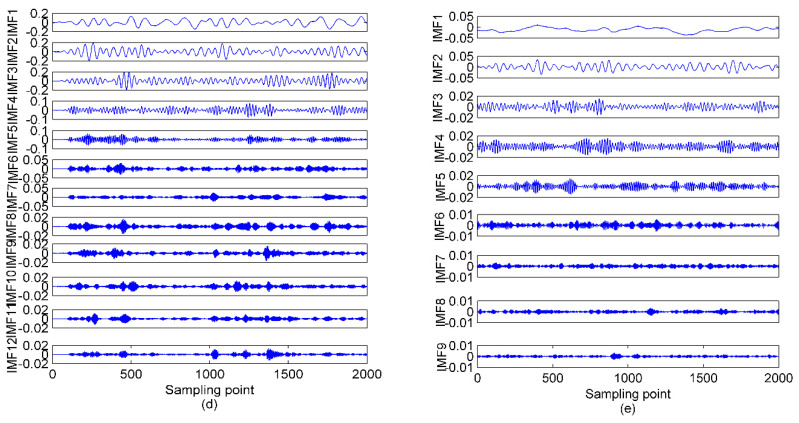
The OVMD of the S−RN signals; (**a**) Ship I; (**b**) Ship II; (**c**) Ship III; (**d**) Ship IV; (**e**) Ship V.

As indicated in Figure 7, for the five types of S-RN signals, the optimal K maximizing the number of IMFs with RWPE greater than 0.2 for the first time is 11, 9, 9, 12, and 9, respectively. The OVMD of the signals are shown in Figure 8.

### 5.2. Recognition of S-RN Samples

**Figure 9:**
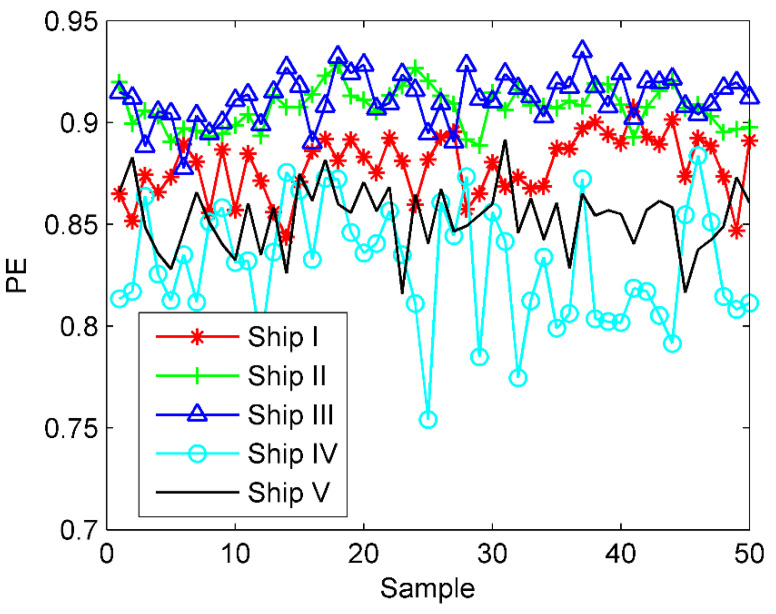
The PE distribution for the five kinds of S−RN samples.

Combined with the time-domain waveforms of the five S-RN signals in Figure 6, due to the pollution of marine environmental noise, the fluctuation of the signal appears to be fairly messy, showing a certain degree of randomness. We can then easily extract the PE of these samples to quantify the uncertainty. The PE distribution of the five types of S-RN samples is presented in Figure 9. As observed from Figure 9, owing to the disturbance of ocean noise, these samples are mixed together and cannot be recognized at all.

For further analysis, Table 5 lists the PE value of the IMF by OVMD with the maximum energy (ME) and that of the IMF with the maximum correlation coefficient (MCC) with the raw S−RN signal. As shown in Table 5, Ships I and III can be well distinguished by these two methods, however, there appear to be relatively close PE values concerning other three categories, which cannot be separated. Then, we employ NSCC to weigh the PE of the IMFs, and the sum of the weighted PE (SWPE) can be obtained, namely:

(18) $$SWPE=\sum_{i=1}^{K}PE_{i}\cdot NSCC_{i}$$

(19) $$NSCC_{i}=SCC_{i}/\sum_{j=1}^{K}SCC_{j}$$

where K is the mode number of OVMD.

**Figure 10:**
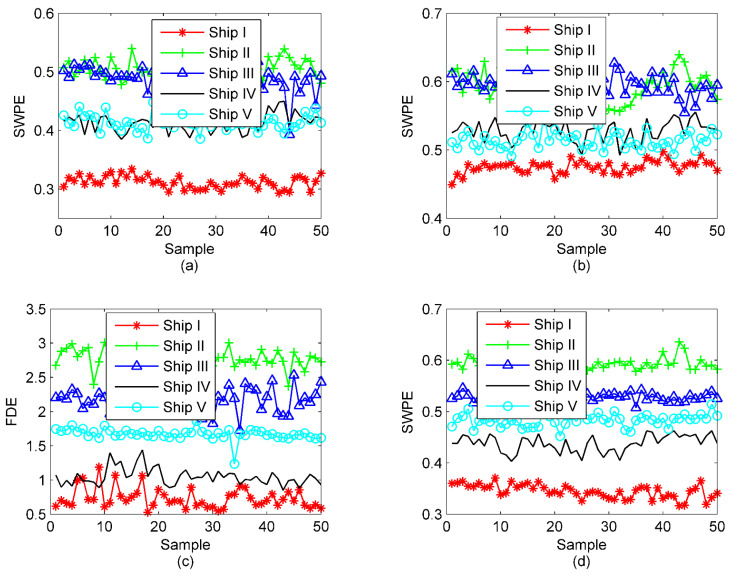
The results under different methods; (**a**) EMD-PE-NSCC; (**b**) EEMD-PE-NSCC; (**c**) VMD-SIMF-FDE; (**d**) the proposed OVMD-PE-NSCC.

For the sake of comparative analysis, the results by EMD-PE-NSCC, EEMD-PE-NSCC, VMD-SIMF-FDE [33], and the proposed OVMD-PE-NSCC are also given in Figure 10.

As in Figure 10a,b, obviously, despite a large number of crossed samples between the other categories, Ship I can be clearly identified, and the overall separation has been improved a little compared with the state without any processing. In terms of VMD-SIMF-FDE, a majority of the samples have become easily recognized with a greater degree of clustering and separation between classes. In marked contrast, intuitively, the proposed OVMD-PE-NSCC outperforms others regardless of the degree of intra-class aggregation and inter-class separation. In order to facilitate the comparison accuracy, 30 randomly selected individuals in each class are utilized for training the SVM multi-class classifier, and the rest are for prediction. The outputs of the classification are displayed in Table 6.

Based on Table 6, the proposed method is significantly superior to other algorithms with higher recognition rate. Our method is an effective tool, the low-frequency IMFs are covered, and concurrently, the recognition rate is also enhanced.

## 6. Conclusions

In order to extract useful features from S-RN signals, a novel technique fully integrating OVMD, PE, and normalized SCC is put forward in this paper. The main innovations and contributions of this paper are as follows:

(1) Compared with PE, AKPE, AAPE, and WPE, the simulation experiments indicate that RWPE, which incorporates amplitude and distance information, is the most competent in distinguishing different signals. Hence, it is innovatively used to lock the mode number for VMD in this paper. This paper successfully conquers the mode number issue for VMD by RWPE, the experimental results on sinusoidal signals have proved the better decomposition performance of OVMD than that of EMD and EEMD.
(2) Five types of measured S-RN signals are selected to verify the performance of our method. The classification results have illustrated that our method is obviously superior to others.
(3) The proposed method can serve as a supplement to the field of underwater acoustic signal processing, as well as extended to other aspects. In future work, we will try to explore other signal decomposition methods and features to further improve the performance of the system.

## Figures and Tables

**Table 1:** The ratio of the maximum and minimum values of the average values for the entropies in the mutation region and other regions.

Parameter	PE	RPE	AAPE	WPE	RWPE
Ratio	1.0012	1.1861	1.0254	1.5356	46.5582

**Table 2:** The center frequency of IMFs after each decomposition.

K	Center Frequency/Hz												
3	7.40	99.14	366.46										
4	7.36	99.12	253.26	375.79									
5	7.29	99.03	188.41	292.77	433.57								
6	7.26	99.01	183.05	277.44	364.69	446.11							
7	5.43	50.09	100.16	190.48	281.86	366.56	446.95						
8	5.42	50.08	100.08	172.82	235.86	298.41	372.46	449.38					
9	5.42	50.08	100.07	170.95	232.67	292.98	361.94	426.22	477.43				
10	5.42	50.07	99.99	156.58	205.94	258.90	309.17	368.90	428.81	478.57			
11	5.41	50.06	99.92	147.42	193.81	243.19	292.30	347.19	386.00	434.47	480.79		
12	5.41	50.05	99.86	138.44	182.52	226.12	266.20	306.82	352.70	392.00	436.61	481.55	

**Table 3:** The RWPE of IMFs after each decomposition.

K						RWPE of IMFs						
3	0.4387	0.3238	0.0556									
4	0.4419	0.3242	0.0654	0.0557								
5	0.4438	0.3251	0.0699	0.0587	0.0968							
6	0.4441	0.3252	0.0764	0.0616	0.0596	0.1153						
7	0.4539	0.4369	0.3247	0.0682	0.0610	0.0594	0.1167					
8	0.4558	0.4370	0.3253	0.0934	0.0658	0.0652	0.0592	0.1200				
9	0.4560	0.4371	0.3254	0.0968	0.0644	0.0652	0.0609	0.0874	0.1611			
10	0.4568	0.4372	0.3260	0.1327	0.0605	0.0692	0.0647	0.0607	0.0913	0.1630		
11	0.4571	0.4372	0.3266	0.1586	0.0662	0.0703	0.0674	0.0663	0.0572	0.0994	0.1668	
12	0.4574	0.4372	0.3269	0.1882	0.0790	0.0616	0.0681	0.0687	0.0689	0.0584	0.1027	0.1679

**Table 4:** The MAE between the center frequency of IMFs by EMD, EEMD, and OVMD with that of corresponding simulation signals.

Parameter	EMD	EEMD	OVMD
MAE	8.8845	7.3984	0.2267

**Table 5:** The PE value of the IMF by OVMD with the maximum energy and that of the IMF with the maximum correlation coefficient with the raw S-RN signal.

	Ship I	Ship II	Ship III	Ship IV	Ship V
ME	0.2580	0.3665	0.3420	0.3548	0.3642
MCC	0.2580	0.3665	0.4205	0.3548	0.3389

**Table 6:** The outputs of classification under different methods.

Algorithms	Number of Misclassified Samples					Accuracy Rate (%)
	Ship I	Ship II	Ship III	Ship IV	Ship V	
PE	10	11	7	12	8	52
EMD-PE-NSCC	0	12	7	14	11	56
EEMD-PE-NSCC	0	17	2	8	10	63
VMD-SIMF-FDE [33]	6	2	2	1	0	89
OVMD-PE-NSCC	0	0	0	3	3	94

## Data Availability

Data available in a publicly accessible repository that does not issue DOIs.

## Abbreviations

S-RN	ship-radiated noise
FT	Fourier transform
STFT	short-time Fourier transform
WT	wavelet transform
EMD	empirical mode decomposition
IMF	intrinsic mode function
EEMD	ensemble empirical mod decomposition
VMD	variational mode decomposition
PE	permutation entropy
WPE	weighted permutation entropy
AAPE	amplitude-aware permutation entropy
RPE	reverse permutation entropy
RWPE	reverse weighted permutation entropy
MPE	multi-scale permutation entropy
FDE	fluctuation-based dispersion Entropy
OVMD	optimized variational mode decomposition
NSCC	normalized Pearson correlation coefficient
MAE	mean absolute error
